# Supplementary material for: Epilepsy in a melanocyte-lineage mTOR hyperactivation mouse model: A novel epilepsy model
Source: PLoS One. 2020 Jan 24;15(1):e0228204. doi: 10.1371/journal.pone.0228204 (PMC6980560; doi:10.1371/journal.pone.0228204)
Supplement: Attachment — Submitted filename: Point-by-point response cover letter.docx [file pone.0228204.s002.docx]

Prof. *Giuseppe Biagini*,

Chief of Editor

*PLOS ONE*

November 28th, 2019

Dear Prof. *Giuseppe Biagini*,

Thank you very much for your e-mail of October/22/2019 with regard to our manuscript (PONE-D-19-25631) entitled “Epilepsy in a melanocyte-lineage mTOR hyperactivation mouse model: a novel epilepsy model” together with the comments from the editor and reviewer. We appreciate the editors and reviewers very much for their positive and constructive comments and suggestions on our manuscript. We have revised our manuscript accordingly. The alterations are referred in this response letter.

We hope that the revised manuscript meets with your approval.

Sincerely,

Mari Wataya-Kaneda, M.D., Ph.D.

Department of Dermatology, Course of Integrated Medicine, Graduate School of Medicine, Osaka University, 2-2 Yamadaoka, Suita, Osaka, 565-0872, Japan

E-mail: [mkaneda@derma.med.osaka-u.ac.jp](mailto:mkaneda@derma.med.osaka-u.ac.jp)

Tel: +81 6 6879 3031. Fax: +81 6 6879 3039

**COMMENTS FROM EDITOR:**

**COMMENT: 1. Please ensure that your manuscript meets PLOS ONE's style requirements, including those for file naming.**

RESPONSE: Thank you for your valuable comments. We have revised our manuscript and modified the file naming to meet PLOS ONE’s style requirements.

**COMMENT: 2. Please include captions for your Supporting Information files at the end of your manuscript, and update any in-text citations to match accordingly.**

RESPONSE: Thank you for your helpful comments. We have added caption for our Supporting Information file at the end of the manuscript and insert the in-text citation to match accordingly.

**COMMENT: 3. To comply with PLOS ONE submissions requirements, please provide methods of sacrifice in the Methods section of your manuscript.**

RESPONSE: Thank you for your important comments. As requested, we have added the description of animal sacrifice as indicated below.

(manuscript, Methods section, page 8, line 140-144, in red with underline).

**“**Mice were anaesthetized with a lethal dose of pentobarbital and sacrificed by intracardially perfusion using ice-cold 1% (w/v) sodium sulfide, followed by 4% paraformaldehyde. The brains were removed, post-fixed for 10% formaldehyde overnight and embedded in paraffin or cryoprotected in 30% sucrose/PBS.**”**

**COMMENTS FROM REVIEWER #1:**

**COMMENT: 1. In material and methods, you should precise the total number of animals used in your experiments.**

RESPONSE: Thank you for your careful reading and valuable comment. As requested, we have added the description about the number of mice we used in each experiment in Materials and methods section in red with underline.

**COMMENT: 2.** **“The fluorescence intensity was quantified by ImageJ and showed same changes (Fig. 6C).” Could please explain the method of quantification by ImageJ?**

RESPONSE: Thank you for this important comment. As suggested, we have added the description of quantification method as indicated below.

(manuscript, Results section, page 14, line 261-262, in red with underline).

The fluorescence intensity was quantified by ImageJ within the range of threshold limit (Jensen EC. Quantitative analysis of histological staining and fluorescence using ImageJ. Anat Rec (Hoboken). 2013 Mar;296(3):378-81).

**COMMENT: 3. In Fig. 3A, 3C, 4A and 4C, the scale bars are different in cKO vs WT. Why?**

RESPONSE: Thank you very much for the careful reading of our manuscript. Because the hippocampus is bigger than WT (as shown in Fig 1C), in Figure 3A, 3C and 4C, to put the whole hippocampus of cKO into the same frame size to WT, images of WT and cKO were shown in different magnification with their different scales respectively. And the hippocampal CA1 pyramidal cells of cKO is also a little bit bigger than WT, in Figure 4A, to put the whole hippocampal CA1 pyramidal cell of cKO into the same frame size to WT, images of WT and cKO were shown in different magnification with their different scales respectively.

**COMMENT: 4.** **It would be preferable to have some sentences about electrographic seizures resumed in a single paragraph. Particularly:
• you should deeply precise how their activity different from baseline activity (duration, rhythms, frequency range and amplitude)
• you should also precise how you define the onset of the seizure: shape of the first part of the seizure, occurrence …
• finally, you should explain how you characterize the end of the seizure: return to baseline activity, post-ictal depression...**

RESPONSE: Thank you for your kind advice. According to your suggestion, we have modified the description as indicated below (manuscript, results section, page 9, line 160-166, in red with underline).

To further characterize these episodes, electrocorticographic activity was recorded for 6-12 hours using a digital video-EEG/EMG system (Neuroscience, inc) in cKO mice and control wild-type littermates at 6 weeks of age. Control mice showed well-organized background activity (under 100-µV spikes) during awake and at rest. By contrast, frequent (2~3 times/hour) high-amplitude sharp waves (above 300-µV spikes, over 10 seconds) were observed during awake in the cKO mice, it was accompanied with seizure-like convulsive movements determined by video recording (Fig 1B).

**COMMENT: 5. In results, you reported that “A recent report indicated that the mTOR pathway regulates excitability of the hippocampal network through controlling the excitatory/inhibitory synaptic balance (19). Therefore, we used immunofluorescence staining to examine excitatory neurons (using anti-CaMKII-a antibody) and inhibitory neurons (using anti-Parvalbumin antibody) in the mouse hippocampus (Fig. 3A). Positive cells were counted, and the ratio of excitatory to inhibitory neurons was calculated (Fig. 3B). No significant difference was observed in the excitatory/inhibitory synaptic balance between cKO mice and control littermates (Fig. 3B).” In this regard, I think you should deeply discuss the crucial** **importance of different interneuron subpopulations in seizure initiation and propagation (refer to “de Curtis, M., and Avoli, M. (2016). GABAergic networks jump-start focal seizures. Epilepsia”), but also in seizure modulation (refer to "Khoshkhoo, S., Vogt, D., and Sohal, V. S. (2017). Dynamic, Cell-Type-Specific Roles for GABAergic Interneurons in a Mouse Model of Optogenetically Inducible Seizures. Neuron").** **Moreover, only specific interneuron subpopulations but not others seem to be involved in the anticonvulsant effects in other models of seizures (refer to “Lucchi et al. (2017). Involvement of PPARγ in the anticonvulsant activity of EP-80317, a ghrelin receptor antagonist. Frontiers in Pharmacology”.)** RESPONSE: Thank you for this helpful advice. According to your suggestion, we have added these three references (25-27) to our manuscript and modified the description as indicated below (manuscript, results section, page 11, line 203-211, in red with underline). “A recent report indicated that the mTOR pathway regulates excitability of the hippocampal network through controlling the excitatory/inhibitory synaptic balance(24). Therefore, we used immunofluorescence staining to examine excitatory neurons (using anti-CaMKII-a antibody) and inhibitory neurons (using anti-Parvalbumin antibody) in the mouse hippocampus (Fig 3A). Positive cells were counted, and the ratio of excitatory to inhibitory neurons was calculated (Fig 3B). No significant difference was observed in the excitatory/inhibitory synaptic balance between cKO mice and control littermates (Fig 3B). It suggests there might have some other players involved in seizure initiation and propagation, e.g. different interneuron subpopulations (25-27).”

**COMMENT: 6. In the discussion, the comparison between mTOR and other markers of neuronal activation used in literature should be done. For instance, you could refer to “Yutsudo et al. (2013). fosB-null mice display impaired adult hippocampal neurogenesis and spontaneous epilepsy with depressive behavior. Neuropsychopharmacology” and to “Giordano et al. (2016). Progressive seizure aggravation in the repeated 6-hz corneal stimulation model is accompanied by marked increase in hippocampal p-ERK1/2 immunoreactivity. Frontiers in Cellular Neuroscience”.**

RESPONSE: Thank you for this valuable comment. we have added these three references (52, 53) to our manuscript and modified the description as indicated below (manuscript, discussion section, page 16, line 304-307, in red with underline).

“Candidate regulators that have emerged in recent years include mTOR, as well as FosB(52), p-ERK1/2(53), tropomyosin-related kinase B, brain-derived neurotrophic factor, Zn^2+^-dependent cascades, and neuron-restrictive silencer factor/repressor element 1-silencing transcription factor pathways(51).”

**COMMENTS FROM REVIEWER #2:**

**COMMENT: In the current Ms, the authors investigate further their melanocyte-lineage mTOR hyperactivation mouse model and try to describe it as a new model of epilepsy. This Ms provides very important data to the field of epilepsy research.**

RESPONSE: Thank you for your careful reading and thank for your interest and compliments on our study.

**COMMENT: However, after reading the title of this Ms, I was quite disappointed when I realised that the authors did not try any classical antiepileptic drug on their supposedly new model of epilepsy. Has this been considered?**

RESPONSE: Thank you for your kind comment. We have tried to treat our mice using the classical mTOR inhibitor-Rapamycin, after 3 weeks of oral rapamycin treatment, epilepsy phenotype was dramatically improved both in frequency and duration of seizures (Fig 6A), however, we have not treated with some other classical clinical antiepileptic drugs. We would like to investigate that in our future study and report that in our next paper.

**COMMENT: I have some other concerns listed below.**

**A) The “Materials and Methods” section lacks sufficient information.**
RESPONSE: Thank you for your careful reading and valuable comment. As requested, we have modified our manuscript according to your suggestions.

**COMMENT: 1. This is not easy to find the number of animals used for each protocol.**

RESPONSE: Thank you for your careful reading and valuable comment. As requested, we have added the description about the number of mice we used in each experiment in Materials and methods section in red with underline.

**COMMENT: 2.  I could not find a clear description of how the animals were sacrified or anesthetized. This comment applies for almost all sections. P5 Ln84 “Histology and immunohistochemistry analyses”: How animals are sacrificed before the brains being fixed? P6Ln95: “Timm staining”: How were the rats anesthetized?** **P6LnLn100: “Electron microscopy examination”: How were the rats sacrificed?**

RESPONSE: Thank you for your important comments. As requested, we have added the description of animal sacrifice as indicated below.

(manuscript, Methods section, page 8, line 140-144, in red with underline).

**“**Mice were anaesthetized with a lethal dose of pentobarbital and sacrificed by intracardially perfusion using ice-cold 1% (w/v) sodium sulfide, followed by 4% paraformaldehyde. The brains were removed, post-fixed for 10% formaldehyde overnight and embedded in paraffin or cryoprotected in 30% sucrose/PBS.**”**

**COMMENT: 3. P7LnLn110: “Primary culture of hippocampal pyramidal cells from adult mice”: How were the slices cut?**

RESPONSE: Thank you for your important comments. As requested, we have added the description about the slice cut as indicated below.

(manuscript, Methods section, page 7, line 106-116, in red with underline).

“Primary neuronal cells were obtained from the hippocampus of 4-week-old wild-type and mutant mice (n = 5 mice/goup) as reported previously(13). Briefly, the hippocampus was dissected and sliced into 0.5-mm sections using tissue slicer (Dosaka microslicer, Kyoto, Japan), removing the dentate gyrus to eliminate granule cells. The sections were digested with papain (2 mg/mL, Worthington, #LS003119 in HA-Ca, BrainBits LLC) at 30°C for 30 min. Cells were released by gentle trituration with a Pasteur pipette. Finally, primary neurons were separated using density-gradient centrifugation (OptiPrep, AXS, #1114542, XX). Cells were cultured in NeurobasalA/B27 medium (Invitrogen, #10888022 and #17504044) with L-Gin (Invitrogen, #25030149), growth factors (5 ng/mL mouse FGF2, Invitrogen, #PMG0034; 5 ng/mL mouse PDGF-BB, Invitrogen, #PMG0044), and gentamycin (Wako, #078-06061) for 1 week before the experiments.”

**COMMENT: 4. P7Ln122: Measurement of [Ca2+]i: Please could you check the composition of your ACSF? What was the osmolarity of this solution?**

RESPONSE: Thank you for your careful reading and the important comment. Regret for this mistake, as suggested, we have added the reference (14) in our manuscript and revised our mistake in the concentration of NaCl (revised 45mM to 127 mM NaCl).

(manuscript, Methods section, page 7, line 121-126, in red with underline).

[Ca^2+^]_i_ in single cells was detected on the basis of fura-2 fluorescence intensity, as reported previuosly(14). Briefly, neurons grown on coverslips were rinsed twice with artificial cerebrospinal fluid (ACSF; 127 mM NaCl, 1.5 mM KCl, 26 mM NaHCO_3_, 1.24 mM KH_2_PO_4_, 10 mM glucose, 1.4 mM MgSO_4_, 2.4 mM CaCl_2_; SIGMA) and incubated at 37°C for 45 min in the presence of fura-2 AM (fura-2 acetoxymethyl ester, DOJINDO, #CS23) with 1.25 mmol/L probenecid (SIGMA) and 0.03% Pluronic® F-127 (SIGMA) in ACSF.

**COMMENT: B) In the discussion at LnP310: “Until now, rodent models of spontaneous recurrent epilepsy have been generated by chemoconvulsants (primarily pilocarpine and kainic acid), neonatal hypoxia, traumatic brain injuries or electrical stimulation(44).” Please note that genetic models of epilepsy are also available to study some types of epilepsy.**

RESPONSE: Thank you for the important comment. According to your suggestion, we have modified the description as indicated below.

(marked manuscript, Discussion section, page 18, line 324-326, in red with underline). “Until now, rodent models of spontaneous recurrent epilepsy have been generated by chemoconvulsants (primarily pilocarpine and kainic acid), neonatal hypoxia, traumatic brain injuries, electrical stimulation or genetic manipulations (55).”
