# Supplementary material for: Epilepsy in a melanocyte-lineage mTOR hyperactivation mouse model: A novel epilepsy model
Source: PLoS One. 2020 Jan 24;15(1):e0228204. doi: 10.1371/journal.pone.0228204 (PMC6980560; doi:10.1371/journal.pone.0228204)
Supplement: Attachment — Submitted filename: Response to reviewers.docx [file pone.0228204.s003.docx]

Prof. *Giuseppe Biagini*,

Chief of Editor

*PLOS ONE*

January 8th, 2020

Dear Prof. *Giuseppe Biagini*,

Thank you very much for your e-mail of December/20/2019 with regard to our manuscript (PONE-D-19-25631R1) entitled “Epilepsy in a melanocyte-lineage mTOR hyperactivation mouse model: a novel epilepsy model” together with the comments from the editor and reviewer. We appreciate the editors and reviewers very much for their positive and constructive comments and suggestions on our manuscript. We have revised our manuscript accordingly. The alterations are referred in this response letter.

We hope that the revised manuscript meets with your approval.

Sincerely,

Mari Wataya-Kaneda, M.D., Ph.D.

Department of Dermatology, Course of Integrated Medicine, Graduate School of Medicine, Osaka University, 2-2 Yamadaoka, Suita, Osaka, 565-0872, Japan

E-mail: [mkaneda@derma.med.osaka-u.ac.jp](mailto:mkaneda@derma.med.osaka-u.ac.jp)

Tel: +81 6 6879 3031. Fax: +81 6 6879 3039

**COMMENTS FROM REVIEWER #1:**

**“The authors have adequately addressed my comments raised in a previous round of review and I feel that this manuscript is now acceptable for publication.”**

RESPONSE: Thank you for your careful reading and valuable suggestions for revising and improving our manuscript.

**COMMENTS FROM REVIEWER #2:**

**“The Ms has improved following the authors's reply to the reviewers's comments. However, in my opinion, supplemental information particularly in the method section, are needed to make this Ms a little bit clearer.”**

RESPONSE: Thank you for your careful reading and important suggestions. We have revised our manuscript according to your comments and suggestions.

**COMMENT: 1. “Primary culture of hippocampal pyramidal cells from adult mice: Please could you precise in which media the slices were cut?”**

RESPONSE: Thank you for your important comments. As requested, we have added the description about the medium as indicated below.

(Manuscript with Track Changes, Methods section, page 7, line 106-111, in red with underline).

“Primary neuronal cells were obtained from the hippocampus of 4-week-old wild-type and mutant mice (n = 5 mice/goup) as reported previously(13). Briefly, the hippocampus was dissected and sliced into 0.5-mm sections in 2 mL HABG medium (40ml HA(Hibernate^TM^-A Medium, Invitrogen, #A1247501; 0.8ml B27, Invitrogen, #17504; 0.1ml L-Glutamine, Invitrogen, #25030081)) at 4°C in a 35-mm-diameter dish using tissue slicer (Dosaka microslicer, Kyoto, Japan), removing the dentate gyrus to eliminate granule cells.”

**COMMENT: 2. “Measurement of [Ca^2+^]i: Please could you precise if the ACSF was "bubbled" with carbogen?”**

RESPONSE: Thank you for your important comments. In our experiments, ACSF has been bubbled with carbogen. As requested, we have added the description as indicated below.

(Manuscript with Track Changes, Methods section, page 8, line 124-129, in red with underline).

“Briefly, neurons grown on coverslips were rinsed twice with artificial cerebrospinal fluid (ACSF; 127 mM NaCl, 1.5 mM KCl, 26 mM NaHCO_3_, 1.24 mM KH_2_PO_4_, 10 mM glucose, 1.4 mM MgSO_4_, 2.4 mM CaCl_2_; SIGMA) and incubated at 37°C for 45 min in the presence of fura-2 AM (fura-2 acetoxymethyl ester, DOJINDO, #CS23) with 1.25 mmol/L probenecid (SIGMA) and 0.03% Pluronic® F-127 (SIGMA) in carbogen-bubbled ACSF.”

**COMMENT: 3. “Animal sacrifice: one can read "Mice were anaesthetized with a lethal dose of pentobarbital and sacrificed by intracardially perfusion using ice-cold 1% (w/v) sodium sulfide, followed by 4% paraformaldehyde." I am a little bit confused. Did the authors perform Primary culture of hippocampal pyramidal cells and measurement of [Ca^2+^]i on cells from animals that have been perfused with 4% paraformaldehyde?”**

RESPONSE: Thank you for your careful reading and the important comment. We have added the information as indicated below.

(Manuscript with Track Changes, Methods section, page 8, line 143-147, in red with underline).

“Mice were anaesthetized with a lethal dose of pentobarbital and sacrificed by intracardially perfusion using ice-cold 1% (w/v) sodium sulfide, followed by 4% paraformaldehyde. The brains were removed for primary culture of hippocampal pyramidal cells, measurement of [Ca2+]i, or post-fixed for 10% formaldehyde overnight and embedded in paraffin or cryoprotected in 30% sucrose/PBS for histologic analyses.”
