# Supplementary material for: Epilepsy in a melanocyte-lineage mTOR hyperactivation mouse model: A novel epilepsy model
Source: PLoS One. 2020 Jan 24;15(1):e0228204. doi: 10.1371/journal.pone.0228204 (PMC6980560; doi:10.1371/journal.pone.0228204)
Supplement: Attachment — Submitted filename: Response to reviewers.docx [file pone.0228204.s004.docx]

Prof. *Giuseppe Biagini*,

Chief of Editor

*PLOS ONE*

January 9^th^, 2020

Dear Prof. *Giuseppe Biagini*,

Thank you very much for your e-mail of January/09/2020 with regard to our manuscript (PONE-D-19-25631R2) entitled “Epilepsy in a melanocyte-lineage mTOR hyperactivation mouse model: a novel epilepsy model” together with the comments from the editor and reviewer. We appreciate the editors and reviewers very much for their positive and constructive comments and suggestions on our manuscript. We have revised our manuscript accordingly. The alterations are referred in this response letter.

We hope that the revised manuscript meets with your approval.

Sincerely,

Mari Wataya-Kaneda, M.D., Ph.D.

Department of Dermatology, Course of Integrated Medicine, Graduate School of Medicine, Osaka University, 2-2 Yamadaoka, Suita, Osaka, 565-0872, Japan

E-mail: [mkaneda@derma.med.osaka-u.ac.jp](mailto:mkaneda@derma.med.osaka-u.ac.jp)

Tel: +81 6 6879 3031. Fax: +81 6 6879 3039

**COMMENTS FROM EDITOR:**

**“Thank you for submitting your manuscript to PLOS ONE. After careful consideration, we feel that it has merit but does not fully meet PLOS ONE’s publication criteria as it currently stands. Specifically, I invite you to complete and correct description of statistical analysis.”**

RESPONSE: Thank you for your important suggestions. We have carefully checked our manuscript again and corrected descriptions of statistical analysis in our revised manuscript.

**COMMENT: 1. “results of statistical analysis are not detailed in results, and should be better illustrated in legends to figures”**

RESPONSE: Thank you for your kind advice. As requested, we have added the detail in legends to figures as indicated below.

(Manuscript with Track Changes, Figure Legend section, page 24-26, line 488-549, in red with underline).

## **“Figure 1. Deletion of *Tsc2* resulted in epilepsy in *Tsc2^Mitf-M^* cKO mice without obvious histoarchitectural changes.**

A*.* Images captured from video recordings, showing typical spontaneous epilepsy in a 6-week-old cKO mouse. B. EEG and EMG segments (300 s) showing normal electrography in a control (WT) mouse and typical electrographic epilepsy in a cKO mouse. C. Relative brain and body weight in cKO mice compared with control (WT) mice at 9 and 11 weeks of age. **p* < 0.05 and ***p* < 0.01 versus WT mice, n = 5 in each group, unpaired *t*-test. D. Hematoxylin staining of murine brain tissue sections, Scale bars: 600 µm. Sizes of hippocampus, Cerebral cortex and Whole brain are shown in the right panel, ***p* < 0.01 versus WT mice, n = 5 in each group, unpaired *t*-test. Data in C and D are expressed as mean ± SD.

## **Figure 2. Hyperactivation of mTOR induced neural excitation in *Tsc2^Mitf-M^* cKO mice.**

A. Histoimmunostaining of whole brain sagittal sections from control (WT) mice (left panels) and cKO mice (right panels) at 5 weeks of age. p-S6 (upper panels) and c-FOS (bottom panels). The black rectangle outlines the area of hippocampus, cerebral cortex, and thalamus, and the detail is shown in the corresponding bottom panels. The circle shows representative p-S6 cytoplasmic and c-FOS nuclear positive staining. Scale bars: large bars, 600 µm; smaller bars, 200 µm. B. p-S6 and c-FOS positive rates (p-S6 or c-Fos-positive neuron cells versus all neuron cells) in the hippocampus, cerebral cortex, and thalamus. Data in C and D are expressed as mean ± SD. **P*<0.05 versus WT mice, n = 5 in each group, unpaired *t*-test.

## **Figure 3. Histopathological analyses of the hippocampal region in *Tsc2*^Mitf^ CKO mice.**

A. Immunofluorescence staining showing excitatory (CaMKII-α) and inhibitory (Parvalbumin) neurons in the hippocampus. The insets show higher magnification of positive cells (arrowheads). B. Numbers of CaMKII-α-positive cells and Parvalbumin-positive cells were double-blind counted in 10 random fields per tissue section. Ratio of inhibitory to excitatory neurons were calculated (n = 5 mice). Data in B are expressed as mean ± SD. n.s. means no significance versus WT mice, unpaired *t*-test. C. Timm staining. The amount of mossy fiber sprouting is similar in cKO and control (WT) mice. Scale bars: A, 200 µm; C, 200 µm**.** n.s., not significant.

## **Figure 4. Hyperproliferation of mitochondria in the neurons of *Tsc2*^Mitf^ CKO mice.**

A. Morphologic examination of p-S6 high-expressed hippocampal CA1 pyramidal cells by electron microscopy. Enlarged cell bodies and mitochondria were observed in the neurons of cKO mice. Bottom panels represent high-magnification images of the regions designated by squares. B. Quantification of mitochondria. The number of mitochondria increased more than 5-fold and the mitochondrial size increased more than 2-fold in neurons from cKO mice, compared with control (WT) mice. (n = 20 cells/mouse, 3 mice in each group). Data in B are expressed as mean ± SD, unpaired *t*-test versus WT mice. C. Immunofluorescence staining showed hyperactivation of mTOR (p-S6) with hyperproliferation of mitochondria (COXIV) in the hippocampus of cKO mice. Scale bars: A upper panel, 2 µm; A bottom panel, 500 nm; C, 200 µm.

## **Figure 5. Neurons from *Tsc2^Mitf -M^* cKO mice were more excitable than neurons from control mice.**

A. Neurons were isolated from 4-week-old mice and cultured for 1 week. Immunofluorescence staining indicates that more than 80% of the isolated cells were neurons (GFAP, astrocytes; MAP2, neurons). B. Calcium imaging of cultured neurons, with corresponding traces shown at the bottom. Neurons from cKO mice respond to particularly low (10 mM) K^+^ stimulation. Scale bars: A, 100 µm; B, 100 µm. WT, wild-type.

## **Figure 6. Rapamycin treatment reduced seizures and number of mitochondria.**

A. Frequency and duration of seizures (sz) in cKO mice in the absence or presence of rapamycin, n = 5 in each group. Data in A are expressed as mean ± SD. Unpaired *t*-test versus vehicle-treated mice. B. Histoimmunostaining analyses of mTOR signaling (p-S6), mitochondria (COXIV), and neuronal excitation (c-FOS). Scale bars: 100 µm. WT, wild-type. C. The fluorescence intensity was quantified by ImageJ. n = 5 in each group. Data in C are expressed as mean ± SD. One-way ANOVA test, followed by Dunnett's post hoc test for multiple comparisons (WT mice versus cKO mice; cKO mice versus Rapamycin-treated cKO mice (cKO_Rapa); WT mice versus cKO_Rapa mice) was performed and adjusted *P* values were calculated. **p* < 0.05, ***p* < 0.01.

# **Supplementary information**

## **S1 video.** Video recording of typical epilepsy.”

**COMMENT: 2. “in methods, I read that you used "two-way analysis of variance", but the post hoc test is not indicated.”**

RESPONSE: Thank you for your careful reading and the important comment. Regret for this mistake, in our experiment, in Figure 6C, the differences of WT mice versus cKO mice, cKO mice versus Rapamycin-treated cKO mice, and WT mice versus cKO_Rapa mice were calculated using One-way ANOVA test, followed by Dunnett's post hoc test. As suggested, the description have been corrected in our revised manuscript as indicated below.

(Manuscript with Track Changes, Figure Legend section, page 26, line 537-547, in red with underline; Methods section, page 9, line 148-152, in red with underline).

## “**Figure 6. Rapamycin treatment reduced seizures and number of mitochondria.**

Frequency and duration of seizures (sz) in cKO mice in the absence or presence of rapamycin, n = 5 in each group. Data in A are expressed as mean ± SD. Unpaired *t*-test versus vehicle-treated mice. B. Histoimmunostaining analyses of mTOR signaling (p-S6), mitochondria (COXIV), and neuronal excitation (c-FOS). Scale bars: 100 µm. WT, wild-type. C. The fluorescence intensity was quantified by ImageJ. n = 5 in each group. Data in C are expressed as mean ± SD. One-way ANOVA test, followed by Dunnett's post hoc test for multiple comparisons (WT mice versus cKO mice; cKO mice versus Rapamycin-treated cKO mice (cKO_Rapa); WT mice versus cKO_Rapa mice) was performed and adjusted *P* values were calculated. **p* < 0.05, ***p* < 0.01.”

(Manuscript with Track Changes, Figure Legend section, page 26, line 537-547, in red with underline; Methods section, page 9, line 148-152, in red with underline).

“**Statistical analyses**

Data are presented as mean ± SD. Unpaired Student’s *t*-test (Microsoft Excel; Microsoft Corp., Redmond, WA) was used for comparisons between two groups. One-way ANOVA test, followed by Dunnett's post hoc test was used for multiple comparisons (Microsoft Excel). *P*-values <0.05 were considered statistically significant.”
